# A Methodology for Extracting Power-Efficient and Contrast Enhanced RGB Images

**DOI:** 10.3390/s22041461

**Published:** 2022-02-14

**Authors:** Elias Dritsas, Maria Trigka

**Affiliations:** Department of Computer Engineering and Informatics, University of Patras, 265 04 Patras, Greece; trigka@ceid.upatras.gr

**Keywords:** histogram equalization, color system, power consumption, smart devices

## Abstract

Smart devices have become an integral part of people’s lives. The most common activities for users of such smart devices that are energy sources are voice calls, text messages (SMS) or email, browsing the World Wide Web, streaming audio/video, and using sensor devices such as cameras, GPS, Wifi, 4G/5G, and Bluetooth either for entertainment or for the convenience of everyday life. In addition, other power sources are the device screen, RAM, and CPU. The need for communication, entertainment, and computing makes the optimal management of the power consumption of these devices crucial and necessary. In this paper, we employ a computationally efficient linear mapping algorithm known as Concurrent Brightness & Contrast Scaling (CBCS), which transforms the initial intensity value of the pixels in the YC_b_C_r_ color system. We introduce a methodology that gives the user the opportunity to select a picture and modify it using the suggested algorithm in order to make it more energy-friendly with or without the application of a histogram equalization (HE). The experimental results verify the efficacy of the presented methodology through various metrics from the field of digital image processing that contribute to the choice of the optimal values for the parameters a,b that meet the user’s preferences (low or high-contrast images) and green power. For both low-contrast and low-power images, the histogram equalization should be omitted, and the appropriate *a* should be much lower than one. To create high-contrast and low-power images, the application of HE is essential. Finally, quantitative and qualitative evaluations have shown that the proposed approach can achieve remarkable performance.

## 1. Introduction

The penetration of smart devices into their users’ daily lives (irrespective of age) is now prevalent. There is no doubt that such devices have been a necessary part of the professional and personal lives of their owners. Due to the limited size and capacity of the batteries, the efficient and effective use of energy is critical to the functionality and usability of the mobile device. These devices include an operating system with advanced computing ability and connectivity. High-speed data access is provided via Wifi and mobile broadband services [1].

The power consumption of a mobile device depends on many factors [2]. These include the technical characteristics of the device, environmental conditions, and user’s behavior separately. The main sources of energy consumption associated with the user’s activities of such smart devices include video and voice calls, sending and receiving messages, browsing the World Wide Web, audio and video streaming.

The usage patterns of a smart device are accountable for the underlying power consumption. Making a voice call requires loading the call application, selecting from the contact list, and dialing the number. Points that are taken into account in the assessment of energy consumption are the duration of the call, the time of accepting the call, and the time of connection. However, more energy consuming is video calling. Many components of the mobile device (such as CPU, display, wireless interfaces) work together to load a web page, which consumes power to achieve the purpose (page loading). Other energy-intensive activities include Google map (CPU, screen, Wifi, 5G, or GPS web mapping application), Google talk (CPU, screen, and audio instant messaging application), YouTube (Web-based video sharing application CPU, monitor, Wifi, 5G), and notifications from social media.

In [3], the authors attempted to determine the most important factors describing the overall power consumption during video streaming, and in [4], they aim to optimize the power consumption of mobile devices for video streaming over 4G LTE networks. Modern devices have various built-in Wireless Network Interfaces (Bluetooth, 4G LTE/5G, Wifi) [5] whose power consumption represents a significant portion of the total power of the system, even when idle. The user activities are also connected with the use of screen, the memory, and CPU [6] and device sensors (e.g., GPS, camera) [7,8].

The management of CPU power consumption is subject to many hardware interventions rather than software. The CPU can save energy by running fewer applications on the device. The memory component (RAM) and the storage media (e.g., SDCard) consume very little energy if they are not enabled for reading/writing files or video streaming. The power consumption on the part of the CPU and RAM is affected not only by the benchmark (gzip, equake, etc.) that is executed but also by the frequency operation. In fact, the higher the frequency, the more energy is expended on both of these components, where between them, the CPU consumes the most power significant difference from RAM [2]. In addition, another significant effect on the energy consumption of a mobile device is the mode in which the device operates. Specifically, in case the mobile device is in idle mode and the screen brightness is off (i.e., the backlight off), the power consumption of the display is significant and third in ranking after graphics and GSM. In this mode, the device is fully awake, but no application is running while the monitor subsystem is in on mode [9]. The power consumed by the screen of a mobile device depends on the screen brightness level and the pixel brightness level of images reflected on it. In particular, the screen energy consumption depends exponentially on the brightness of the image pixels, but linearly on the screen brightness level [10].

Organic Light Emitting Diodes (OLED) displays have become prevalent in modern electronic devices due to the power saving they offer compared to their predecessors, Liquid Crystal Displays (LCD) and Light Emitting Diodes (LED) [11]. The power required to display content on OLED depends mainly on it, regardless of the screen brightness level (white-maximum screen brightness level and a black screen-zero screen brightness level) [12]. Dash and Hu [13] experimentally confirmed that the content displayed on such screens has a significant effect on power consumption. The pixel values of the images that users set as a background on their device, even with the minimum screen brightness, play an important role in the power consumed by the screen and therefore in the total power consumed by the device. Since the screen consumes enough of the total power of the mobile device, it is desirable to develop an image processing algorithm, which is able to save energy in the display panel (screen). It is the topic that will concern us in the following sections. In particular, we aim to process the candidate wallpapers to reduce the energy required for their display and enhance their contrast.

The problem of power saving in OLED displays has been approached in the literature in various ways for either images or videos. There are methods that tackle the problem as power constrained and contrast enhancement using histogram-relevant priors, while most recent works employ Sparse Coding-based techniques [14,15]. With the advent of Artificial Intelligence (AI) and Machine Learning (ML), an unsupervised CNN-based [16] method has been suggested to produce high perceptual quality and less power consumption.

The present work was based on backlight-scaling techniques for LCDs and the need to reduce the power consumption on content-dependent displays, such as OLED, by content enhancement using the YC_b_C_r_ model and the luminance part Y of the displaying image following the philosophy [17]. We adopt different processing functions, namely, linear transformation and then adaptive histogram equalization of Y instead of the sigmoid function that the authors employ in [17]. Here, we will present a computationally simple algorithm that implements a linear transformation of the input image luminance pixels to simultaneously affect the contrast and brightness so that the resulting image consumes less energy in its display and keeps its quality at an acceptable level. The extracted image will have low contrast, since the linear scaling limits the dynamic range. Therefore, histogram equalization is applied to obtain high-contrast images. Such a choice may depend on the user preferences.

The structure of the paper is organized as follows. Section 2 presents the histogram technique and equalization process. Moreover, it describes in detail the proposed methodology, the performance metrics, and the power-constrained perspective of our approach. Section 3 describes the results and performance evaluation of the suggested method in terms of power savings and image quality under the involved parameters. Section 4 discusses the elaborated method in relation to similar studies with the same objective and outlines some future directions of the current research. Finally, Section 5 concludes the paper.

## 2. Materials and Methods

The elaborated approach will be founded on the histogram of a digital image. Therefore, in the next section, we will present some background knowledge about this technique. In addition, our methodology will be described, and the definition of some useful metrics for the evaluation part will be given.

### 2.1. Histogram

The histogram shows the distribution of data and, in image processing, it is used to show the distribution of pixel values in an image. Usually, we normalize the histogram by dividing each value by the total number of pixels in the image, let *n*. Then, the normalized histogram is given by the function $p_{k}=\frac{n_{k}}{n}$, for k=0,1,…,L−1 levels of gray, where $L=2^{l}$ (l=8 bits), and we could say that it gives an approximation of the probability of occurrence of each level of gray.

The histogram of a digital image with gray levels in the interval [0,L−1] is a discrete function that expresses the number of pixels of the image in each gray level. In addition, it is a technique that can describe and demonstrate the contrasts of an image and achieve contrast improvements through histogram modification techniques. Contrast expresses the differences in brightness between light and dark areas of a natural scene. Brightness is a factor that affects the overall view of an image and refers to its overall brightness or darkness. Contrast stretching is an image enhancement technique that improves the contrast of an image by “extending” its pixel range to [0,L−1].

Histogram modification techniques are classified into linear, non-linear, and adaptive [18]. Linear and non-linear transform pixel values using linear and nonlinear mathematical functions, respectively. In these techniques, histogram matching or histogram specification is also included. Some traditional techniques of these categories are presented in Table 1.

From adaptive techniques, the most representative is the histogram equalization. This method smooths the contrasts in an image either locally or globally. The global histogram equalization is applied to the whole image simultaneously. The local equalization scans the image using an overlapping sliding window (small or medium). It applies global equalization to the window transforming the central pixel. Moreover, to transform the boundary pixels, it is necessary to apply padding with an appropriate number of the symmetric values of the image on the perimeter. On the other hand, to reduce the time and computational complexity of the implementation, the boundary pixels of the image remain usually unprocessed. It is characterized by high computational complexity that is enhanced in large images.

The histogram equalization is a histogram specification technique in which the specified histogram is uniformly distributed. In [19], the authors suggest a different histogram specification method in which the brightness and contrast are tuned by adjusting the shape of the probability density function (pdf) of the 1D and 2D Gaussian distribution using the mean and variance of the histogram of the original image.

The histogram equalization can be applied to a variety of color systems [20,21,22], such as RGB, LAB, YIQ, YC_b_C_r_, HSV, and HSI. The images to be processed are in the RGB color system and then transformed into one of the rest of the color systems. Histogram equalization is usually applied to the intensity channel. Nonetheless, if it is applied to the chromatic components, the chromaticity of the new image will differ from the original [23]. The histogram equalization is applied to the components L, Y, V, and I, respectively, and the image is reconstructed in the current color system. Finally, it is restored to the original RGB color system, and thus, the new enhanced contrast RGB image is recovered. Some techniques based on histogram equalization [24] are the following:

- Brightness Preserving Histogram Equalization with Maximum Entropy;
- Brightness Preserving Bihistogram Equalization (BPHE);
- Bihistogram Equalization (BBHE);
- Dualistic Subimage Histogram Equalization (DSHE);
- Recursive Mean-Separate Histogram Equalization;
- Minimum Within-Class Variance Multi-Histogram Equalization (MWCVMHE);
- Minimum Middle Level Squared Error Multi-Histogram Equalization (MMLSEMHE).

### 2.2. Proposed Approach

The proposed approach aims to save display power by reducing the image illumination while preserving its quality through brightness and contrast control (namely, linear transformation) and histogram equalization. The ultimate goal of this work is to reduce the battery power consumption of a mobile device in order to increase its lifespan.

#### 2.2.1. Description

Users select an image of their choice as wallpaper on their device screen. The energy consumed by the screen is related to the brightness and color values of the pixels in the image. In [25], color transformation algorithms are studied for the purpose of replacing colors (background/foreground, theme) in mobile GUIs, with new ones that consume less energy. The results show that the proposed solutions in [25] can save energy over 75% with acceptable quality results. They were tested on OLED screens. Lee et al. in [26] suggest a new power management scheme for GUI applications with multi-color objects in OLED-based mobile devices adapting colors based on Euclidean distance in CIELAB color space and mobile power budget. These algorithms focus on “creating” new, less energy-intensive colors, while our approach leaves colors unaffected and focuses on brightness and contrast. Since white consumes the maximum and black consumes the least energy, we will be able to reduce image energy by reducing its brightness. The proposed solution attempts to reduce the energy consumed by the screen of the smart device in order to reduce the total energy it consumes. Specifically, the basis of our proposal is a linear and then adaptive transformation of the pixels’ intensity values to eliminate the contrasts and reduce the overall brightness of the candidate wallpaper. The darker the image, the less energy the device’s screen will consume to display it.

#### 2.2.2. The Main Steps of the Algorithm

1. Reads the RGB input image I.
2. Converts the image from the RGB to YC_b_C_r_ and retrieves the three components $Y,C_{b},C_{r}$.
3. Applies the Concurrent Brightness & Contrast Scaling technique to the intensity component Y (not the color components). It is a linear function of the form $Y_{CBCS}=aY+b$, where $Y_{CBCS}$ captures the intensity pixel values of the transformed image and Y denotes the intensity of the original image. We should find the appropriate a>0 and *b* that reduce the image energy without compromising its quality. Parameter *a* adjusts the contrast, and parameter *b* adjusts the image brightness.
4. Checks if the resulting values of step 3 are between 0 and 255. If it is below 0, they are set to 0, while if it is above 255, they are set to 255.
5. The component $Y_{CBCS}$ (after the previous transformation) is additionally subjected to a global histogram equalization that eliminates the contrasts.
6. Replaces the values of the component Y with the ones that emerged from the previous step, reassembles image components in YC_b_C_r_, and returns the processed image to RGB.
7. Estimates the energy consumed by the new image, which is expected to be lower than the original, and evaluates the quality of the resulting image through the Peak Signal-to-Noise Ratio (PSNR), Mean Squared Error (MSE), and Structural Similarity Index (SSIM) performance metrics.

**Figure 1:**
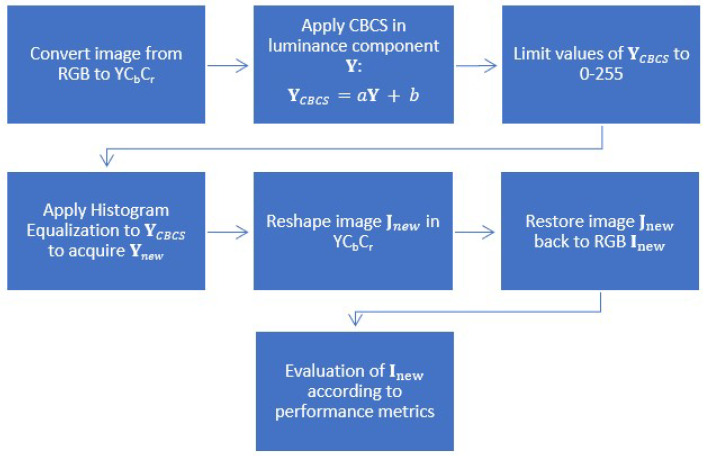
Block diagram of the proposed approach.

The above steps are also illustrated in Figure 1.

#### 2.2.3. Histogram Equalization of $Y_{CBCS}$ with and without Power Constraint

Here, we will present the main steps of the histogram equalization technique as described by Lee et al. in [27]. It should be noted that this technique will be applied to the linearly transformed luminance component denoted as $Y_{CBCS}$.

Let h be the column vector whose elements $h_{k}$ are the number of pixels corresponding to the intensity *k*. The probability of the intensity *k*, $p_{k}$, is defined as $p_{k}=\frac{h_{k}}{1^{T}h}$. The cumulative distribution function (CDF) of the intensity *k* is given as

(1) $$c_{k}=\sum_{i=0}^{k-1}p_{i}.$$

The histogram equalization corresponds to the input pixel at a new value for the output pixel so that the histogram of the new image is uniform. The $y_{k}$ transformation function corresponds to the intensity *k* of the input image at the value $y_{k}$ in the output image. For a *l*-bits image, the transformation function is as follows:

(2) $$y_{k}=\lfloor \left(2^{l}-1\right)c_{k}+0.5\rfloor ,k=0,1,\ldots ,2^{l}-1.$$

Ignoring the rounding operator, and combining (1), (2) then the following relation for 8-bit images is obtained:

(3) $$y_{k}-y_{k-1}=255p_{k},\;0<k\leq 255,\;y_{0}=255p_{0}.$$

Equations (2) and (3) can be summarized to

(4) $$Dy=\bar{h},$$

(5) $$D=\left[\begin{array}{ccccccc} 1 & 0 & 0 & \ldots & 0 & 0 & 0\\ -1 & 1 & 0 & \ldots & 0 & 0 & 0\\ \vdots & \vdots & \ddots & \vdots \\ 0 & 0 & 0 & \ldots & -1 & 1 & 0\\ 0 & 0 & 0 & \ldots & 0 & -1 & 1 \end{array}\right].$$

The normalized vector $\bar{h}=255\frac{h}{1^{T}h}$.

It is known that the power of an image is mainly defined by Y. Incorporating the power model into the proposed technique, we enhance both the contrast by equalizing the histogram of the linearly transformed component $Y_{CBCS}$ (aY+b) and save power (by reducing the histogram values for large intensities of Y). The Lagrangian multiplier technique is employed to compromise between these two goals.

Histogram equalization can be performed by solving $Dy=\bar{h}$ or equivalently minimizing ${∥Dy-\bar{h}∥}^{2}$, while power consumption can be saved by decreasing $y^{T}Hy$. Therefore, contrast enhancement and power saving can be achieved simultaneously by minimizing the sum of these two terms. Then, the Lagrangian cost function is given by

(6) $$C\left(y\right)={\left(Dy-\bar{h}\right)}^{T}\left(Dy-\bar{h}\right)+\lambda y^{T}Hy,$$

where λ is a Lagrangian multiplier and $H=diag(h_{0},h_{1},\ldots ,h_{255})$. By differentiating the cost function C(y) with respect to y and setting it to 0, the power constrained solution is

(7) $$y={\left(D^{T}D+\lambda H\right)}^{-1}D^{T}\bar{h}.$$

The proposed methodology is based on the case where λ=0. In this case, the equalized gray levels y are estimated by

(8) $$y=D^{-1}\bar{h}.$$

The y captures the new intensity levels of equalized component $Y_{CBCS}$ so that the transformed image consumes less energy for its display and has uniformly distributed contrast. The solution in (7) depends on the values of the parameter λ, which controls the contribution of the power term and the visual quality of the resulting image. Since the two terms in (6) have different orders of magnitude, the parameter λ should be the inverse order of magnitude of $y^{T}Hy$ to avoid suppressing the contrast enhancement processing by the power saving term and achieve a better power saving with acceptable quality loss. The dominance of the power term will make the image very dark and thus lower energy. However, the resulting image will be perceptually indistinguishable from the original. The elaborated methodology is also presented in the algorithmic form in Algorithm 1.
**Algorithm 1** HE-CBCS.1:**Input** Read the input image I2:**Input** Convert to YC_b_C_r_: J=rgb2ycbcr(I);3:Y=J(:,:,1), $C_{b}=J\left(:,:,2\right)$, $C_{r}=J\left(:,:,3\right)$;4:**Input** Read the set of parameters a,b;5:**Output** New RGB image $I_{new}$6:**for**p=1 to |a| **do**7:   **for** q=1 to |b| **do**8:     a=a(p), b=b(q), $Y_{CBCS}=aY+b$;9:     Limit $Y_{CBCS}$ into [0,255];10:     Apply HE through (4) or (7) in $Y_{CBCS}$ and restore y;11:     Each pixel intensity in Y is replaced by the intensity levels of y to acquire $Y_{new}$;12:     Reshape image in YC_b_C_r_: $J_{new}$ = reshape($Y_{new},C_{b},C_{r}$);13:     Convert $J_{new}$ from YC_b_C_r_ to RGB: $I_{new}=ycbcr2rgb\left(J_{new}\right)$;14:     Estimate metrics: $ssim\left(p,q\right)=SSIM\left(I,I_{new}\right)$, $psnr\left(p,q\right)=PSNR\left(I,I_{new}\right)$;15:     $mse\left(p,q\right)=MSE\left(I,I_{new}\right)$, $prr\left(p,q\right)=PRR\left(I,I_{new}\right)$;16:   **end for**17:**end for**

### 2.3. Image Quality Evaluation Metrics

In this section, we demonstrate the most common loss functions [28,29,30] that are utilized to capture the difference between the original (I) and the reconstructed (J) images.

MSE is the squared Euclidean (L_2_) distance that measures the degradation of image quality compared to the original image. Mathematically, it is defined as:

(9) $$MSE=\frac{\sum_{i=1}^{M}\sum_{j=1}^{N}{\left(J_{i,j}-I_{i,j}\right)}^{2}}{MN},$$

where M,N are the dimensions of the image.

PSNR gives the quality of the reconstructed image after processing. The higher the value, the better the quality of the resulting image. Mathematically, it is defined as:

(10) $$PSNR=20log\left(\frac{255}{\sqrt{MSE}}\right).$$

For color images, the values of MSE and PSNR are calculated as the average of the values for each component R,G,B separately.

SSIM is a metric that evaluates the similarity between two images. It is the most reliable indicator of how close is the processed image to the original. It receives values in the range of [0,1] and the closer the two images are, the closer to one the values of this index will be. According to [31,32], its general math type adjusts the importance of the components that capture the luminance and contrast comparison, respectively. A specific type for the SSIM is

(11) $$SSIM\left(I,I_{new}\right)=\frac{\left(2\mu_{I}\mu_{I_{new}}+C_{1}\right)\left(2\sigma_{I,I_{new}}+C_{2}\right)}{\left(\mu_{I^{2}}+\mu_{I_{new}}^{2}+C_{1}\right)\left(\sigma_{I}^{2}+\sigma_{I_{new}}^{2}+C_{2}\right)}$$

where $\mu_{I}$ and $\mu_{I_{new}}$ are the average intensity of input image I and output image $I_{new}$, respectively, $\sigma_{I}$ and $\sigma_{I_{new}}$ are the standard deviation of I and $I_{new}$, respectively, $\sigma_{I,I_{new}}$ is the co-variance of I, and $I_{new}$, $C_{1}$ and $C_{2}$ are small constants.

### 2.4. Power Rating Metrics

The power consumption of an OLED panel is based on the metric:

(12) $$Power=\omega_{0}+\sum_{i=1}^{N}\sum_{j=1}^{M}\left(\omega_{R}R_{i,j}^{\gamma }+\omega_{G}G_{i,j}^{\gamma }+\omega_{B}B_{i,j}^{\gamma }\right),$$

where N,M denote the width and height of the panel, respectively, and $R_{i,j},G_{i,j},B_{i,j}$ are the red, green, and blue intensities of the (i,j)-th pixel, respectively. The coefficients γ and $\omega_{R},\omega_{G},\omega_{B}$ are display-dependent and 2≤γ≤3 [33]. Alternatively, the power can be approximated by using only the Y-component in the YC_b_C_r_ color space as follows:

(13) $$Power=\sum_{i=1}^{N}\sum_{j=1}^{M}Y_{i,j}^{\gamma },$$

where $Y_{i,j}$ is the intensity of the Y-component of the (i,j)-th pixel.

A key metric for the evaluation is the power reduction rate (PRR) of the transformed image, which is defined as follows

(14) $$PRR=\left(1-\frac{P_{final}}{P_{original}}\right)\times 100\%.$$

If this metric takes negative values, it means that the processing has increased the energy of the image. We are looking for that set of parameters that reduces energy but also maintains the quality of the image at acceptable levels for the user.

**Remark** **1.** *Taking into account that $Y_{CBCS}=aY+b$ and the dependence of power in (13) by intensities $Y_{i,j}$, the PRR is rewritten as*

(15) $$\begin{array}{cc} PRR & =\left(1-\frac{\sum_{i=1}^{N}\sum_{j=1}^{M}{\left(aY_{i,j}+b\right)}^{\gamma }}{\sum_{i=1}^{N}\sum_{j=1}^{M}Y_{i,j}^{\gamma }}\right)\times 100\%\\ \; & =\left(1-\sum_{i=1}^{N}\sum_{j=1}^{M}{\left(\frac{aY_{i,j}+b}{Y_{i,j}}\right)}^{\gamma }\right)\times 100\%. \end{array}$$

*To achieve the desired goal $\frac{aY_{i,j}+b}{Y_{i,j}}\ll 1$ or $\left(a-1\right)Y_{i,j}+b\ll 0$. Given that $Y_{i,j}>0$, 0<a<1 and $b\ll \left(1-a\right)Y_{i,j}$ should hold.*

## 3. Results

### 3.1. Experiments Environment and Data

**Figure 2:**
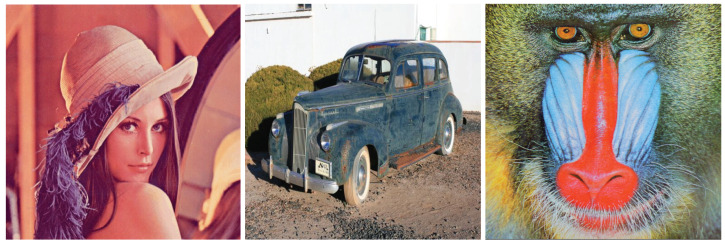
Data for evaluation: Lena, Car, and Baboon RGB images.

The environment in which experiments were carried out has the following characteristics: Intel(R) Core(TM) i7-9750H CPU @ 2.60GHz 2.59 GHz 16 GB Memory, Windows 10 Home, 64-bit Operating System, x64-based processor, and Matlab 2019a. The results reported here concern Lena, Baboon, and Car (MATLAB demo images) color images of size 512 by 512 pixels. The color depth is 24 bits, i.e., eight bits per color channel in the range of 0 to 255. In addition, to estimate the power consumption of an OLED panel, the coefficients have been set as $\left(\gamma ,\omega_{0},\omega_{R},\omega_{G},\omega_{B}\right)=\left(2,0,70,115,154\right)$. The input images are shown in Figure 2.

### 3.2. Evaluation

Now, we will describe the evaluation of the proposed approach. The whole processing becomes in component Y [34] since the color and luminance channels, in the YC_b_C_r_ color system, are independent. However, the evaluation metrics are estimated and depicted in the RGB color system since we aim to minimize the total power for displaying a color image keeping its perceptive quality at an acceptable level. Let us recall that *a* defines the contrast and *b* defines the image brightness.

**Figure 3:**
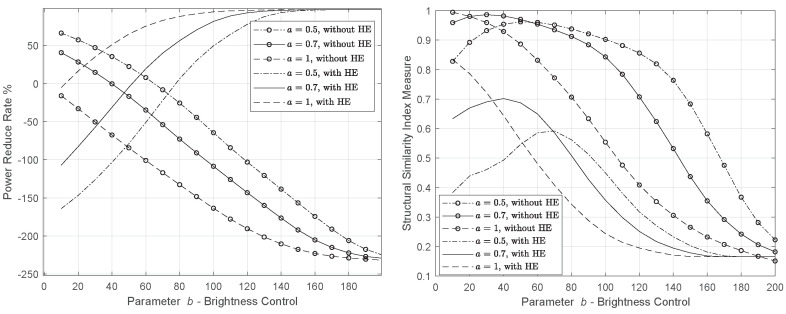
PRR and SSIM of the $Y_{CBCS}$ (without HE) and $Y_{new}$ ($Y_{CBCS}$ with HE) for Lena image.

**Figure 4:**
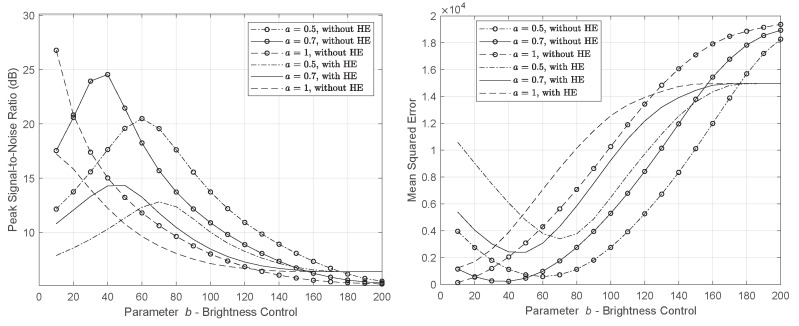
PSNR and MSE of the $Y_{CBCS}$ (without HE) and $Y_{new}$ ($Y_{CBCS}$ with HE) for Lena image.

Figure 3 and Figure 4 illustrate the power reduction rate and the impact of the processing in the Lena image. We evaluate the CBCS method with and without the application of histogram equalization. Through the experiments, we found that the appropriate values of the parameters a,b that make the PRR positive maximize the SSIM and PSNR, and minimize the MSE are in the range 0.5≤a≤1, 10≤b≤200, respectively.

**Figure 5:**
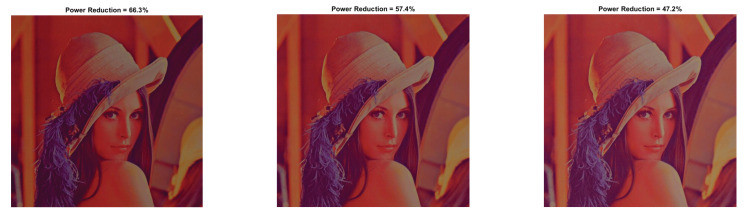
Resulted image $I_{new}$ using $Y_{CBCS}$ (without HE) for a=0.5 and b = [10:10:30].

**Figure 6:**
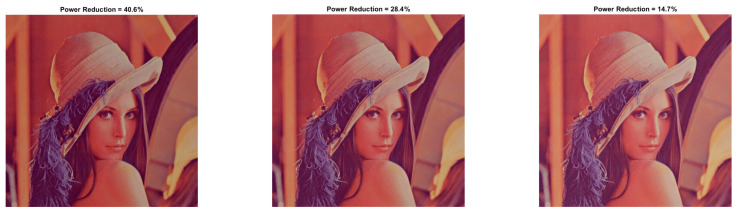
Resulted image $I_{new}$ using $Y_{CBCS}$ (without HE) for a=0.7 and b = [10:10:30].

First, we assess the impact of the CBCS method without the use of HE. According to Figure 3, a=0.5 achieves the best power saving with a satisfactory performance from an image loss quality perspective (as SSIM reveals). In Figure 5 and Figure 6, we depict the output image, and in Table 2 and Table 3, we record the PRR for the three pairs of parameters a,b that achieve the highest PRR and SSIM. We do not report the results for a=1 (and >1), because in this case, for any value of parameter *b*, the PRR is negative and the SSIM is very low.

The CBCS method decreases image power by narrowing the Y dynamic range of gray levels with lower boundary values. This holds when a≪1 and for low values of *b*. Such a technique can save power, but the output image has low contrast.

Focusing on the figure that depicts the PRR, we observe that the histogram equalization plays a decisive role in reducing the image power when a=1 and the parameter *b* that controls the brightness moves from medium to high levels. In addition, PRR curves (without and with the HE application) converge to different points. The opposite behavior is caused by the $D^{-1}$ that affects the gray levels $\bar{h}$ (see Equation (8)). According to (13), the image power depends on $y^{\gamma }={\left(D^{-1}\bar{h}\right)}^{\gamma }$, which, when combined with (14), make the curves with HE converge in different directions.

Considering the power-constrained solution (7) of (6), which tries to both apply histogram equalization and reduce the image power, the value of parameter λ makes dominant the term that captures the $Y_{CBCS}$ power (the linearly transformed luminance Y of input image). From the experiments, we verified that this specific parameter, even when it takes values at the level of $10^{-4}$, makes the minimization of the cost function in (6) independent of the parameters a,b and the performance metrics behavior is flat. Specifically, it significantly decreases the image power by 96%, but it simultaneously deteriorates the image quality, as expected.

**Figure 7:**
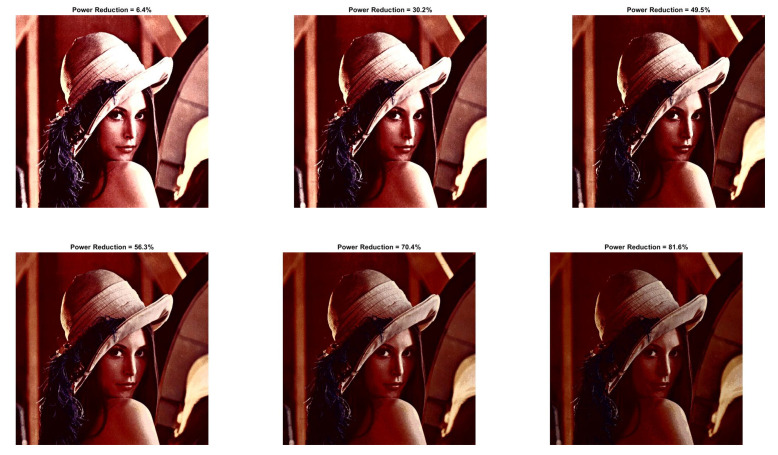
Lena resulted image $I_{new}$ for a={0.5,0.7}, *b* = [80:10:100].

**Figure 8:**
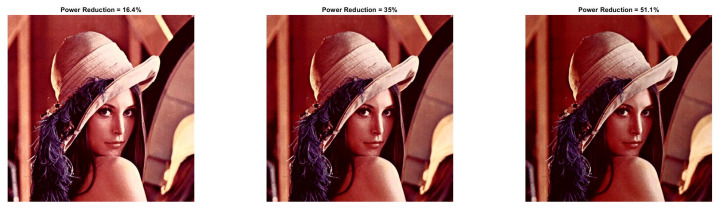
Lena resulted image $I_{new}$ for a=1 and *b* = [20:10:40].

In Table 4 and Figure 7 and Figure 8, the results concern the case of HE without the power constraint solution (λ=0), making the underlying problem controllable by parameters a,b. In particular, it is observed that if parameter *a* that controls the image contrasts is equal to 0.5 (see Figure 3) and parameter *b* (brightness) is between 10 and 70, the processing increases the pixels’ power, making the image quite bright. As *b* takes higher values and, especially for b≥80 (see Figure 3 and Figure 7), the image power is reduced, which is reflected as the darkening of the image. The same process is repeated for a=0.7 where higher power reduction is observed at the cost of lower visual quality. In Figure 7 and Figure 8, we observe that for a=1,b=20, a power decline by 16.4% is achieved, contrary to a=0.5, where b=80 is needed to reduce the image power by at least 6.4% (approximately 3 times lower than a=0.5).

From Table 4, we see that when *a* is doubled, namely a=1 (b=90), the total power savings become three times greater than a=0.5 (b=90), at the cost of a half drop in similarity. Hence, maintaining brightness and enhancing image contrast can achieve power saving. Nonetheless, the optimal parameters should meet both the power and quality requirements. The highest PRR (16.4%) was recorded in SSIM=0.786 where a=1,b=20. This outcome is also depicted in Figure 8.

To reinforce the validity of the methodology, we present the quantitative and qualitative results for two more images (Baboon and Car). Specifically, we chose to present the visual results for those parameters where the images’ power is reduced.

In Table 5 and Table 6, we summarize the Baboon image research outcomes without the application of HE for a=0.5,0.7, respectively. In Table 7 and Table 8, we outline the Car’s image performance metrics in the same case.

**Figure 9:**
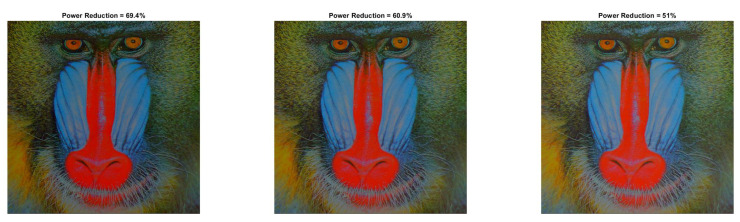
Baboon resulted image $I_{new}$ using $Y_{CBCS}$ (without HE) for a=0.5 and *b* = [10:10:30].

**Figure 10:**
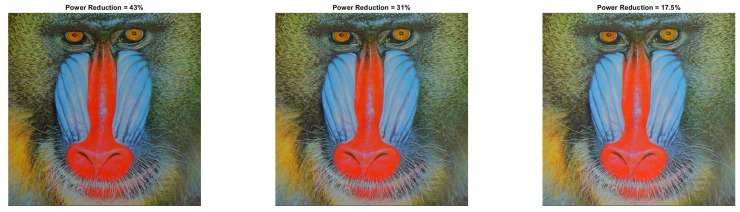
Baboon resulted image $I_{new}$ using $Y_{CBCS}$ (without HE) for a=0.7 and *b* = [10:10:30].

**Figure 11:**
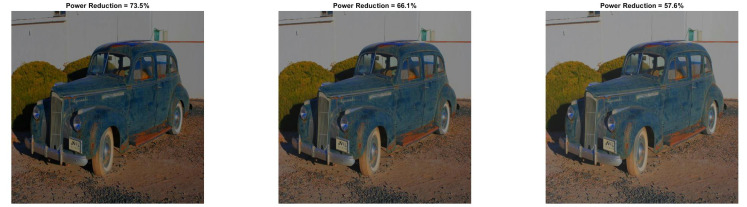
Car resulted image $I_{new}$ using $Y_{CBCS}$ (without HE) for a=0.5 and *b* = [10:10:30].

**Figure 12:**
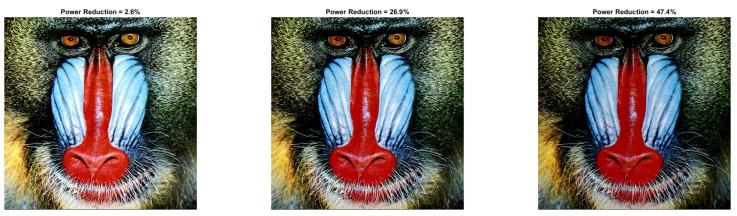
Car resulted image $I_{new}$ using $Y_{CBCS}$ (without HE) for a=0.7 and *b* = [10:10:30].

Figure 9 and Figure 10 show the visual impact of the processing without HE. Moreover, Figure 11 and Figure 12 depict the visual effects on Car image. In both these images (as in Lena), if a=1, the image power increases. Again, we observe that a=0.5 is suitable for power reduction with slightly lower SSIM values than Lena.

In Table 9 and Table 10, we summarize the Baboon and Car outcomes after the application of HE. In Baboon, for a=1 and b=10, a PRR of 2.63% is achieved with SSIM 0.742. Considering the same parameters in Lena’s image, the power increases. However, in Car’s image, a significant power reduction of 25.34% is achieved, which shows that the performance varies depending on the input image.

**Figure 13:**
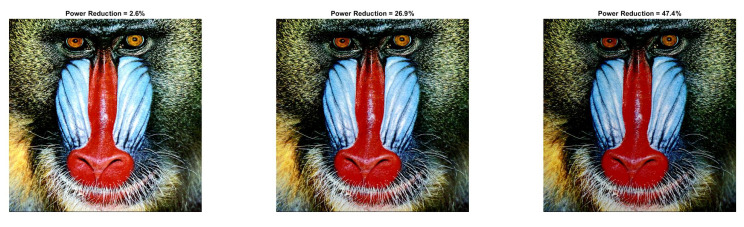
Baboon resulted image $I_{new}$ using $Y_{CBCS}$ (with HE) for a=1 and *b* = [10:10:30].

**Figure 14:**
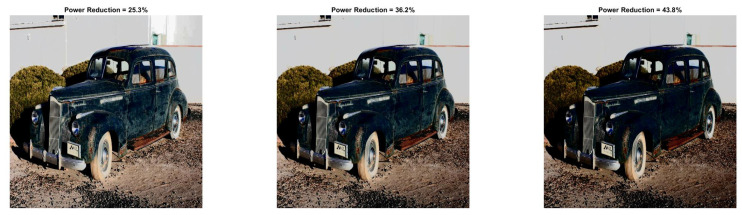
Car resulted image $I_{new}$ using $Y_{CBCS}$ (with HE) for a=1 and *b* = [10:10:30].

In Figure 13 and Figure 14, we illustrate the processed images after HE. As is expected, high-contrast and low-power images are derived. We would like to note that similar curves as the ones depicted in Figure 3 and Figure 4 are derived for Baboon and Car images. We omitted them since their behavior is similar to Lena. Finally, the application of the power-constrained model in these images has the same impact as Lena.

## 4. Discussion

Most approaches focus primarily on wireless interfaces and device sensors generally in an effort to manage the issue of energy saving. Our proposal presents a different approach to the issue of energy saving in modern mobile devices.

The histogram can be a useful tool for producing images with improved contrast and brightness as well as low power. In the literature, several solutions have been proposed that use existing histogram modification techniques (linear, nonlinear, adaptive) to produce images with improved contrast and less power for their display. The so-called Power Constrained Contrast Enhancement (PCCE) employs a nonlinear luminance transformation to reduce OLED power and improve contrast [27,35,36,37]. PCCE performs an unconstrained optimization of power and contrast controlled by a single parameter, while the Low-Overhead Adaptive Power Saving and Contrast Enhancement (LAPSE) [38] minimized power under a mean SSIM (MSSIM) constraint. Comparing our proposal with LAPSE, although they have a similar goal, the luminance transformation of each pixel is based on $Y_{i,j}^{new}=a_{3}Y_{i,j}^{3}+a_{2}Y_{i,j}^{2}+a_{1}Y_{i,j}+a_{0}$, which is a more general polynomial function of cubic order. Notice that setting $a_{3},a_{2}=0$, the luminance processing function is simplified or reduced to our proposal. Moreover, both in our work and LAPSE, it utilized SSIM as the key metric to determine the optimal parameters. However, the main target of the latter’s algorithm (LAPSE) is time overhead. Recent work in [39] produces contrast-enhanced images applying a modified Land-Effect method that uses white balance and retinex filter. The resulting image has visually better color contrasts than the original and saves power by 13.16%.

Recent works on power saving in OLED displays adopt the Sparse Representations theory in the power-constrained model. In [14,15], the authors propose a mixed-norm Power-Constrained Sparse Representation (PCSR) model where the processing is applied at the patch level. Their method is evaluated using VIF (Visual Information Fidelity) with satisfactory performance for power levels ranging from 40% to 70%. From this perspective, if we apply our HE-based approach inside a patch, the global histogram equalization is equivalent to local. Then, this procedure is repeated iteratively to all image patches. In [16], differently from the current and previous works, Yin et al. exploit the deep CNN learning network to produce power-saved images. The input image is transformed into YUV color space, and the visual quality is assessed through various metrics such as SSIM, VIF, etc., for 10%, 20%, and 30% power reduction.

The elaborated approach is founded on the well-known CBCS algorithm that processes the luminance of each pixel of the image linearly, rendering the brightness and contrast of the enhanced image user-controlled. By incorporating this step prior to histogram equalization, the image processing becomes parametric-dependent and makes the power minimization task controllable as well. We choose to do the processing in the YC_b_C_r_ color system for specific reasons. First of all, YC_b_C_r_ is a family of color systems commonly used for the digital representation of images and videos in digital photo-systems (DSP). The simplicity of the transformation and the immediate decoupling of the luminance from the color components make this color space attractive. Moreover, when a still RGB image is being prepared for encoding under the jpeg encoding and compression system, it is transformed into the YC_b_C_r_ color system, where the component Y keeps the luminance information [38]. Another reason is that the jpeg standard has been proven to be more energy-efficient among other frequently used ones, and thus, our idea could contribute to even more energy-friendly jpeg images.

In addition, now, the intensity transformation is treated as a linear regression problem. The new intensity is estimated based on one previous weighted value of the corresponding pixel intensity (this weight captures the image contrast) and a parameter that captures the total brightness. Then, the resulted intensity is equalized to preserve image quality and reduce image display power simultaneously. Inspired by this analogy, (traditional) data-driven regression methods from statistical signal processing (such as Auto Regressive—AR, Moving Average—MA, Exponentially Weighted Moving Average—EWMA) and machine or deep learning techniques could be evaluated as challenging alternatives for the prediction of the new intensity component Y. Our purpose is to thoroughly investigate the discussed frameworks in [40,41] and formulate the appropriate methodology that best suits our objectives. In addition, the pixels in a predefined neighborhood around the candidate pixel could be considered to estimate the new intensity value. However, this would increase the complexity of the problem (the number of the involved parameters), making its tracking more complex.

Our proposal could concern not only the candidate wallpapers on a user’s device but also general-purpose (or application-independent) images, making them even more energy efficient by reducing their brightness. A positive aspect, in this case, is that these images could be part of websites, so we may achieve energy savings on the part of the browser, which, as we presented above, spends a significant amount of the total energy and slows down battery life. The future direction of this work is to investigate the above processing on video frames to reduce the display energy during playback and incorporate more effective contrast enhancement techniques into the formulation.

## 5. Conclusions

In conclusion, in this paper, we proposed a methodology for extracting power-efficient images while maintaining the perceptual quality at an acceptable level. To validate the suggested algorithm, a quantitative and qualitative analysis was adopted that revealed the optimal parameters under which the processed image exhibited the lowest levels of distortion and simultaneously consumed lower power. In the context of the qualitative analysis, the visual results aim to illustrate the impact of the proposed algorithm. The experimental results showed that without histogram equalization, the parameter *a* should be much lower than one to save power, but low-contrast images are generated. By integrating the histogram equalization step, the parameter *a* should be equal to one in order to achieve a balance between the user’s goals, i.e., a high-contrast image with less visual distortion and lower power.

## Figures and Tables

**Table 1:** Linear and nonlinear histogram modification techniques where $0=f_{min}<g<f_{max}=L-1$.

Linear	Nonlinear
Contrast Stretching g(f)=af+b, $\left[s_{1},s_{2}\right]\to \left[t_{1},t_{2}\right]$ $a=\frac{\left(t_{2}-t_{1}\right)}{\left(s_{2}-s_{1}\right)}$, $b=t_{1}-s_{1}a$	Logarithmic Function g(f)=blog(af+1)
$g\left(f\right)=\frac{f-f_{k}}{f_{k+1}-f_{k}}\left(g_{k+1}-g_{k}\right)+g_{k}$$f_{k}<f\leq f_{k+1}$, $f_{k},g_{k},k=0,1,\ldots ,K-1$	Exponent Function $g\left(f\right)=be^{af-1}$
Thresholding $g\left(f\right)=g_{0}$, f≤T $g\left(f\right)=g_{1}$, f>T	Power Law $g\left(f\right)=af^{k}$ k<1 reinforce dark areas k>1 suppress light areas k=2 square law–exponent function k=3 cubic law–logarithmic function
Multi-level thresholding $g\left(f\right)=g_{k},f_{k}<f\leq f_{k+1}$	-

**Table 2:** Lena image without HE for a=0.5.

	b=10	b=20	b=30
a=0.5 PRR	66.26	57.42	47.16
a=0.5 SSIM	0.828	0.893	0.932
a=0.5 PSNR	12.15	13.75	15.59

**Table 3:** Lena image without HE for a=0.7.

	b=10	b=20	b=30
a=0.7 PRR	40.59	28.37	14.75
a=0.7 SSIM	0.959	0.980	0.986
a=0.7 PSNR	17.55	20.59	23.94

**Table 4:** Lena image performance metrics with HE for a={0.5,0.7,1} and *b* = [10:10:100].

	b=10	b=20	b=30	b=40	b=50	b=60	b=70	b=80	b=90	b=100
a=0.5 PRR	−164.07	−146.75	−126.29	−103.37	−78.71	−50.99	−22.12	6.39	30.22	49.52
a=0.5 SSIM	0.383	0.440	0.461	0.493	0.546	0.586	0.592	0.562	0.513	0.450
a=0.5 PSNR	7.88	8.58	9.38	10.30	11.33	12.33	12.82	12.37	11.25	10.02
a=0.7 PRR	−107.06	−82.69	−57.47	−31.19	−4.79	19.55	40	56.35	70.42	81.61
a=0.7 SSIM	0.634	0.670	0.691	0.702	0.687	0.650	0.586	0.509	0.428	0.357
a=0.7 PSNR	10.81	12.07	13.32	14.29	14.34	13.26	11.78	10.46	9.36	8.47
a=1 PRR	−5.14	16.40	35.03	51.05	64.97	82.92	88.08	91.65	94.09	95.42
a=1 SSIM	0.834	0.786	0.722	0.644	0.560	0.481	0.408	0.344	0.289	0.244
a=1 PSNR	17.24	15.85	13.95	12.23	10.78	9.64	8.75	8.07	7.54	7.14

**Table 5:** Baboon image without HE for a=0.5.

	b=10	b=20	b=30
a=0.5 PRR	69.45	60.94	51.04
a=0.5 SSIM	0.730	0.805	0.856
a=0.5 PSNR	11.78	13.31	15.12

**Table 6:** Baboon image without HE for a=0.7.

	b=10	b=20	b=30
a=0.7 PRR	43.02	30.98	17.54
a=0.7 SSIM	0.928	0.956	0.967
a=0.7 PSNR	17.09	20.12	23.93

**Table 7:** Car image without HE for a=0.5.

	b=10	b=20	b=30
a=0.5 PRR	73.53	66.15	57.61
a=0.5 SSIM	0.747	0.813	0.853
a=0.5 PSNR	10.52	11.76	13.14

**Table 8:** Car image without HE for a=0.7.

	b=10	b=20	b=30
a=0.7 PRR	46.27	35.84	24.33
a=0.7 SSIM	0.932	0.949	0.954
a=0.7 PSNR	15.71	17.99	20.38

**Table 9:** Baboon image performance metrics with HE for a={0.5,0.7,1} and *b* = [10:10:100].

	b=10	b=20	b=30	b=40	b=50	b=60	b=70	b=80	b=90	b=100
a=0.5 PRR	−178.89	−160.38	−138.18	−112.14	−84.79	−56.67	−28.41	−1.16	26.20	52.88
a=0.5 SSIM	0.416	0.456	0.495	0.536	0.560	0.556	0.523	0.479	0.428	0.358
a=0.5 PSNR	7.44	8.16	9.06	10.17	11.24	11.86	11.75	11.16	10.35	9.38
a=0.7 PRR	−112.55	−86.69	−60.85	−33.42	−5.83	21.36	46.41	66.59	81.07	90.13
a=0.7 SSIM	0.612	0.641	0.649	0.634	0.602	0.549	0.474	0.386	0.292	0.207
a=0.7 PSNR	10.63	11.92	12.95	13.30	12.83	11.88	10.65	9.38	8.23	7.32
a=1 PRR	2.63	26.95	47.36	63.62	76.34	85.53	91.29	94.55	96.33	97.27
a=1 SSIM	0.742	0.678	0.596	0.502	0.403	0.307	0.224	0.159	0.114	0.085
a=1 PSNR	15.30	13.71	11.97	10.44	9.16	8.13	7.36	6.81	6.42	6.14

**Table 10:** Car image performance metrics with HE for a={0.5,0.7,1} and *b* = [10:10:100].

	b=10	b=20	b=30	b=40	b=50	b=60	b=70	b=80	b=90	b=100
a=0.5 PRR	−100.17	−83.08	−63.47	−44.05	−26.61	−11.24	2.24	13.48	21.81	27.34
a=0.5 SSIM	0.663	0.691	0.712	0.715	0.689	0.634	0.562	0.488	0.418	0.355
a=0.5 PSNR	9.53	10.86	12.63	14.43	15.30	14.81	13.53	12.12	10.86	9.91
a=0.7 PRR	−46.05	−31.09	−16.59	−3.18	8.42	17.89	36.75	50.14	54.22	57.45
a=0.7 SSIM	0.796	0.791	0.760	0.702	0.624	0.538	0.433	0.386	0.330	0.294
a=0.7 PSNR	15.09	16.90	17.40	16.16	14.29	12.56	11.12	9.97	9.28	8.83
a=1 PRR	25.34	36.21	43.78	49.72	54.39	58.15	62.21	67.58	74.47	81.52
a=1 SSIM	0.766	0.685	0.596	0.506	0.425	0.360	0.313	0.279	0.249	0.221
a=1 PSNR	16.54	14.26	12.52	11.18	10.16	9.42	8.87	8.40	7.89	7.34
